# Glaciers and Ice Sheets As Analog Environments of Potentially Habitable Icy Worlds

**DOI:** 10.3389/fmicb.2017.01407

**Published:** 2017-07-28

**Authors:** Eva Garcia-Lopez, Cristina Cid

**Affiliations:** Microbial Evolution Laboratory, Centro de Astrobiología (Consejo Superior de Investigaciones Cientificas-Instituto Nacional de Técnica Aeroespacial) Madrid, Spain

**Keywords:** glaciers, ice sheets, habitability, analog environments, icy worlds, cold adaptation, extremophiles, psychrophiles

## Abstract

Icy worlds in the solar system and beyond have attracted a remarkable attention as possible habitats for life. The current consideration about whether life exists beyond Earth is based on our knowledge of life in terrestrial cold environments. On Earth, glaciers and ice sheets have been considered uninhabited for a long time as they seemed too hostile to harbor life. However, these environments are unique biomes dominated by microbial communities which maintain active biochemical routes. Thanks to techniques such as microscopy and more recently DNA sequencing methods, a great biodiversity of prokaryote and eukaryote microorganisms have been discovered. These microorganisms are adapted to a harsh environment, in which the most extreme features are the lack of liquid water, extremely cold temperatures, high solar radiation and nutrient shortage. Here we compare the environmental characteristics of icy worlds, and the environmental characteristics of terrestrial glaciers and ice sheets in order to address some interesting questions: (i) which are the characteristics of habitability known for the frozen worlds, and which could be compatible with life, (ii) what are the environmental characteristics of terrestrial glaciers and ice sheets that can be life-limiting, (iii) What are the microbial communities of prokaryotic and eukaryotic microorganisms that can live in them, and (iv) taking into account these observations, could any of these planets or satellites meet the conditions of habitability? In this review, the icy worlds are considered from the point of view of astrobiological exploration. With the aim of determining whether icy worlds could be potentially habitable, they have been compared with the environmental features of glaciers and ice sheets on Earth. We also reviewed some field and laboratory investigations about microorganisms that live in analog environments of icy worlds, where they are not only viable but also metabolically active.

## Introduction

Although possible life elsewhere may be different than it is on Earth, several minimum conditions of habitability have been defined: a solvent (water), a source of energy, a group of biologically essential elements (on Earth they are H, C, N, O, S, and P) and some physicochemical conditions (temperature, pH, water activity, etc.) (Hoehler, 2007; Priscu and Hand, 2012; Cockell et al., 2016).

Water on Earth was originated from innumerable collisions with icy comets and asteroids and from volcanic outgassing of the planet’s interior (Madigan et al., 2012). At that time, due to the intense heat, water would have been present only as water vapor. Then, Earth cooled down, forming a solid crust and condensing water into oceans around 4.5 billion years ago (Bada et al., 1994). The presence of liquid water implies that conditions could have been compatible with life within a couple of hundred million years after Earth was formed (Madigan et al., 2012). A similar scenario could be the one observed nowadays in icy worlds from our solar system.

The icy worlds of the solar system and beyond meet these conditions of habitability, but their magnitudes are different. It is necessary to know what the intervals of these values are to make them habitable. To know the limits of life, we must first establish what these limits are on Earth, since it is the only case we know. One of the most life limiting features on Earth is low temperature. Actually, the Earth can be considered a cold place. For instance, 90% of the Earth’s oceans have a temperature of 5°C or less (Russell, 1990). When terrestrial habitats are included, over 80% of the Earth’s biosphere is permanently cold. Among terrestrial environments, 85% of Alaska, 55% of Russia and Canada, 20% of China, and the majority of Antarctica are permanently cold (Pewe, 1995). In our solar system, six of the other eight planets are permanently cold, and hence understanding life’s adaptations to cold environments on our planet should be useful in the search for and understanding of life on other planets (Rodrigues and Tiedje, 2008). Living organisms that inhabit the more extreme environments on Earth are microorganisms. Among them, microorganisms inhabiting glaciers and ice sheets could be those that support the environment more similar to the conditions found in icy worlds.

## The Characteristics of Habitability known for the icy Worlds

Insights returned by the solar system planetary missions, and the research in terrestrial extreme environments have widened the concept of habitability in the universe. Many of the potentially habitable places in the universe have cold temperatures. That is why it is important to know the lower limit of temperature in which life is possible. After the successful missions Galileo and Cassini-Huygens to the Jupiter and Saturn systems respectively, this priority has focused on the research of environments potentially habitable in some icy worlds such as Europa, Enceladus, and Titan (Alcazar et al., 2010). It has been hypothesized that life on Earth could have arrived in a process of lithopanspermia. Similarly, such transfers were most likely to occur when these satellites were warmer and meteorites could reach their liquid inner oceans through a thinner ice shell (Worth et al., 2013). Below the most interesting icy worlds are reviewed (**Table 1**), since they are potentially habitable.

**Table 1:** The characteristics of habitability known for the icy worlds of the solar system.

	Mars	Europa	Ganymede	Callisto	Titan	Rhea	Enceladus	Triton
Solvent	Brines (Martin-Torres et al., 2015)	Briny ocean (Stevenson, 2000)	Water ocean? (Dalton et al., 2010)	Water ocean? (Cooper et al., 2001)	Methane, ethane ocean (Stofan et al., 2007; Béghin et al., 2010)	Water ice (Dalton et al., 2010)	Briny ocean (Kite and Rubin, 2016)	Sub-surface ocean? (Dalton et al., 2010)
Source of energy	Solar radiation (Cockell et al., 2016); Chemical (Westall et al., 2013, 2015)	Cryovolcanism (Dalton, 2010); Hydrothermal processes (Price, 2007)	Cryovolcanism (Schenk et al., 2001)	Cryovolcanism? (Dalton, 2010)	Cryovolcanism (Fortes et al., 2007)	Radioactive elements, tidal interactions (Dalton et al., 2010)	Geochemical (Sekine et al., 2015); Hydrothermal processes (Waite et al., 2017)	Radiogenic heating (Dalton et al., 2010)
Essential elements (identified or predicted)	C, H, N, O, P, S (McGlynn et al., 2012)	C, H, O, S (Dalton, 2010)	C, H, O, S (Calvin et al., 1995; Cooper et al., 2001)	C, H, O, S (Cooper et al., 2001)	C, H, N (Teolis et al., 2010)	C, H, O (Dalton et al., 2010)	C, H, N, O, Ar (Kite and Rubin, 2016)	C, H, N, O (Tyler et al., 1989; Dalton, 2010)
Physico-chemical conditions	Temperature -60°C (Aerts et al., 2014). In winter at the poles -125°C (Forget et al., 1995)	Temperature -187°C to -141°C Possible in interior ocean -3°C (Spencer et al., 1999; Dalton, 2010)	Temperature -183°C to -113°C (Dalton, 2010)	Temperature -193°C to -115°C (Dalton, 2010)	Rainfall, pressure∼Earth (Rahm et al., 2016) Temperature -203°C to -73°C	Temperature -220°C to -174°C (Dalton, 2010)	Temperature -240°C to -50°C (Dalton, 2010)	Temperature -237°C to -234°C (Dalton, 2010)
Habitability potential	Low	High	Low	Low	Low	Low	High	Low


### Mars

Mars can be considered an icy world because of its low temperatures (Aerts et al., 2014). On average, the temperature on Mars is about -60°C (**Table 1**). And, in winter, temperatures can get down to -125°C at the poles (Forget et al., 1995). Several present and future missions -Curiosity, ExoMars, and possibly Mars 2020- have been equipped with the necessary instrumentation to detect habitable environments *in situ* (Westall et al., 2015). The exploration of Mars has revealed some of the atmospheric and surface properties (Westall et al., 2013). Important gasses have been detected in the Martian atmosphere such as carbon monoxide (96%) and nitrogen (1.9%) (Kaplan et al., 1969). Furthermore, there is an increasing number of reports about superficial water in the past. This has been suggested by the numerous alluvial fans observed in craters (di Achille and Hynek, 2010; Hurowitz et al., 2017), and by the detection of phyllosilicate minerals, which likely represent surface weathering profiles produced by aqueous alteration of the basaltic crust (Fairén et al., 2010). The lack of an ozone layer and the low atmospheric pressure on Mars result in an environment with an intense UV radiation. UVC and UVB daily fluence at 200–315 nm on Mars at present day has been calculated as ∼361 kJ/m^2^, while this value is ∼39 kJ/m^2^ on Earth (Cockell et al., 2000). This characteristic along with the low temperature contribute to the biologically inhospitable nature of the present Martian surface (Cockell et al., 2000). However, the existence of traces of methane in the Martian atmosphere has been hypothesized to be a plausible evidence for life (Holm et al., 2015). Recent spacecraft-based and Earth ground-based studies have calculated methane values of 10 – 60 ppbv in the Martian atmosphere (Formisano et al., 2004; Mumma et al., 2009; Geminale et al., 2011; Holm et al., 2015). Afterward, the Curiosity rover registered seasonally and regionally variable values around 0.69 ppbv at Gale crater, with episodically elevated levels reaching 7.2 ppbv (Webster et al., 2015). One of the most important discoveries in the exploration of Mars has been the detection of putative hydrothermal phases, including serpentine and phyllosilicates, a weathering product of rocks due to water-rock interactions (Michalski et al., 2013). Serpentinization, a geological low-temperature metamorphic process in which rocks are oxidized and hydrolyzed into serpentinite, has been proposed as an alternative abiotic source for the observed methane low traces.

### Satellites of Jupiter

Jupiter has 67 known moons with confirmed orbits. Among them, the Galilean moons Europa, Ganymede and Callisto, are the most interesting from an astrobiological point of view (**Table 1**). The main research about the icy satellites’ surface composition have been carried out by the missions Pioneers 10 and 11, Voyagers I and II, Galileo, Cassini and the New Horizons missions (Grundy et al., 2007; Dalton et al., 2010). Future missions such as JUICE (JUpiter ICy moons Explorer) mission from ESA, will perform detailed investigations of Jupiter with a particular focus on the these three satellites.

#### Europa

**FIGURE 1:**
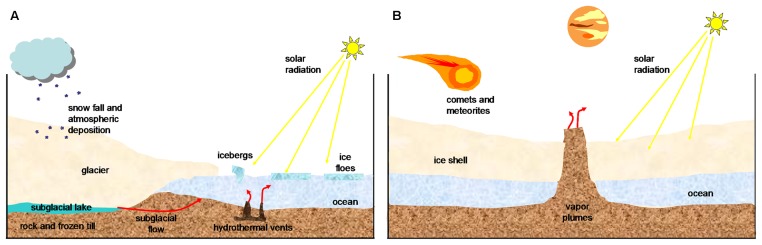
A schematic diagram of cold environments. Surface layers of **(A)** a terrestrial glacier and **(B)** an icy satellite such as Europa. (Adapted from Marion et al., 2003; Priscu and Hand, 2012; Garcia-Lopez and Cid, 2017).

Europa is one of the icy worlds in the solar system where potential habitability is more plausible (**Table 1**). Its surface, perhaps geologically active, presents a rigid icy crust that is up to several kilometers thick (Schmidt et al., 2011). The putative ocean of Europa has drawn a remarkable interest (Chyba, 2000; Grundy et al., 2007), as it could be a similar environment to the hydrothermal vents or to the Antarctic Lake Vostok on Earth (**Figure 1**).

Space missions have obtained some data about its temperature distribution (Spencer et al., 1999) and the radiation at surface (Cooper et al., 2001). Temperature on the surface of Europa is around -180°C, but the possible interior ocean can have a much warmer temperature up to -3°C. Observations from Earth have lead to the suggestions that atmospheric oxygen could come from reactions in which water molecules were broken to give rise to molecular hydrogen and oxygen (Hall et al., 1995; Shematovich and Johnson, 2001); through processes related to serpentinization and ice-derived oxidants. On the icy crust of Europa, phyllosilicates have also been detected (Marion et al., 2003); these minerals are often associated with organic matter on Earth. The possible collision with comets and asteroids could be the source of minerals on Europa’s surface (Chyba and Phillips, 2001).

As mentioned before, the JUICE mission is planned to explore some icy moons and its potential habitability. For Europa, the focus is on the chemistry essential to life, including the characterization of the surface organic and inorganic chemistry, and on understanding the formation of surface features. If life exists in Europa’s ocean, it would be difficult to detect, since it would be necessary to drill the ice before finding the liquid ocean in which the living forms could be preserved. However, it would also be plausible that some convection processes facilitated their rise to the surface (Marion et al., 2003). Life in Europa could exist around hydrothermal vents on the ocean floor. However, some reports have demonstrated that microorganisms could also exist inside ice, as they live in the terrestrial glaciers (Price, 2007). Although temperatures in Europa can be as low as -180°C, the lower limit of temperature at which microbial activity has been detected in laboratory cultures seems to be around -196°C (Junge et al., 2006). Since the environment must be extremely cold and saline, only microorganisms hypothetically analogous to psychrophiles and halophiles could be the ones living in Europa (Marion et al., 2003).

#### Ganymede

Ganymede’s surface has a high number of craters and is mainly composed of silicate rock and water ice. This surface also presents bright, smooth terrains that could be formed by cryovolcanic flows (Schenk et al., 2001). A water ocean (**Table 1**) is believed to exist nearly 200 km below its surface, included between layers of ice (Dalton et al., 2010).

The moon is surrounded by a thin atmosphere composed of O, O_2_, O_3_, and some H_2_ (Calvin et al., 1995; Cooper et al., 2001).

#### Callisto

Callisto is mainly composed of rock and ice. It may contain an ocean of liquid water beneath its cratered surface (**Table 1**). The atmosphere on Callisto is composed of CO_2_ and may probably contain molecular oxygen (Cooper et al., 2001). The likely presence of an ocean within Callisto indicates that it can or could harbor life. Although Callisto is the furthest from the intense radiation of Jupiter, life is considered to be less likely than on Europa.

### Satellites of Saturn

Among the more than sixty Saturn moons, only Rhea and Titan have enough mass to hold an atmosphere. However, Titan’s atmosphere is mostly nitrogen and methane, with only little amounts of O_2_ and CO_2_ (Teolis et al., 2010).

#### Titan

The surface of Titan is thought to be made largely of ice, with oceans of liquid methane and possibly ethane (Stofan et al., 2007; Béghin et al., 2010). Its climate produces wind and rain, which generate fields of dunes in the dark equatorial regions of Titan, rivers, lakes and seas (Rahm et al., 2016) (**Table 1**). Its atmosphere is composed of nitrogen, methane and complex hydrocarbon compounds. The existence of cryovolcanism on the moon’s surface has been suggested (Fortes et al., 2007). Cryovolcanism is a very important phenomenon from an astrobiological point of view, because it allows the mixing of organic molecules such as hydrocarbons, nitriles and cyanides with water, giving rise to oxidized more complex prebiotic molecules (Neish et al., 2006; Marin-Yaseli et al., 2017). Titan appears to be very Earth-like in its geology, despite the very different surface conditions and composition. Complex carbon compounds named tholins exist on Titan, as in comets and in the atmospheres of the outer planets (Dalton et al., 2010). Theoretically, tholins might interact with water in a process of hydrolysis to produce complex molecules similar to those found on the early Earth.

#### Rhea

Rhea is around 527,000 km from Saturn and orbits within its magnetic field. Its average surface temperature is estimated to be -180°C. This moon is characterized by divisions between the leading and trailing hemisphere. The leading hemisphere is uniformly bright, while the trailing hemisphere displays networks of bright stripes on a dark background that overlaps with craters. Cassini observations have established that these lineaments created by tectonic fractures are rich in water ice (Dalton et al., 2010). The moon is surrounded by a thin atmosphere composed of 70% O_2_ and 30% CO_2_. The O_2_ is believed to be formed when water molecules are split by energetic particles in a process of radiolysis (Dalton et al., 2010).

#### Enceladus

Saturn’s moon Enceladus harbors a global ocean of salty water under its icy crust (Kite and Rubin, 2016). Recently, the Cassini-Huygens mission detected significant amounts of hydrogen in the plume, and a warm subsurface region with prominent thermal anomalies that had not been identified before (Le Gall et al., 2017). These observations imply the presence of a broadly distributed heat production and transport system below the south polar terrain with ‘plate-like’ features, and suggest that a liquid reservoir could exist some kilometers beneath the ice crust (Le Gall et al., 2017). From previous flybys, Cassini determined that nearly 98% of the gas in the plume was water, and the rest was a mixture of other molecules, including carbon dioxide, methane and ammonia (Kite and Rubin, 2016). And recent observations made by the Cassini spacecraft, have found molecular hydrogen in the Enceladus plume, an important finding that represents an evidence for hydrothermal processes (Waite et al., 2017). The existence of serpentinization reactions in Enceladus, which would generate hydrogen, has been suggested. Under particular conditions, this hydrogen could supply chemical energy to support chemoautotrophic life (Sekine et al., 2015). A sample return mission to Enceladus has been proposed, Life Investigation For Enceladus (LIFE), in order to study its high astrobiological potential (Tsou et al., 2012).

### Satellites of Neptune

Neptune is one of the coolest places in the solar system. Life is unlikely unless geological activity made it possible. However, its satellites with volcanic activity are interesting for astrobiological exploration.

#### Triton

Voyager 2 discovered that Triton had a volcanic activity, which consists of the melting of ice water and nitrogen, and perhaps methane and ammonia (Tyler et al., 1989). The atmosphere is composed of nitrogen and methane, the same compounds that exist on Saturn’s largest moon, Titan. Nitrogen is also the main compound of the Earth’s atmosphere, and methane on Earth is normally associated with life. Nevertheless, like Titan, Triton is extremely cold. If that were not the case, these two components of the atmosphere would be considered signs of life. However, due to the geological activity and possible internal warming, it has been suggested that Triton could harbor primitive life forms in liquid water below the surface, in the same way that it has been suggested for Jupiter’s moon Europa (Dalton et al., 2010). Evidence of the detection of the HCN ice has also been presented. HCN is a product of the photolytic processing of nitrogen and carbon bearing molecules, and represents an intermediate step in the production of macromolecules of astrobiological interest (Dalton et al., 2010; Marin-Yaseli et al., 2017).

### Habitability Beyond the Solar System. Exoplanets

It is considered that any exoplanet that rotates around its star in an area not too hot and not too cold, allowing the existence of liquid water, could contain some form of life (Kasting et al., 1993). In addition to temperature, another of the conditions of habitability is the radiation emitted by the star. Red dwarfs are considered excellent candidates. These are small stars, with less than half the mass of our Sun and with surface temperatures below 4,000°C (Anglada-Escude et al., 2016). The discovery of thousands of exoplanets that are very different from our own solar system could completely change several aspects of the planetary sciences as we know them today (Seager and Bains, 2015).

Beyond our Solar System, Proxima Centauri b, a planet similar to Earth in size, provides an exciting opportunity to learn about the evolution of terrestrial planets orbiting M dwarfs. This planet is located in the habitable zone away from its star, and might even contain an ocean; thus it has been considered as potentially habitable. However, red dwarfs are also prone to have stellar eruptions much more frequent and powerful than those of our Sun. These eruptions generate bursts of high-energy radiation that break the molecules in their constituent atoms and ionize them; so electrons are easily lost in the space. Over time, positively charged particles are sent away from the surface of the planet (Airapetian et al., 2017). Hydrogen, essential for water and the lightest element, is the most vulnerable to this process. In all likelihood, Proxima Centauri b must have also lost most of its atmospheric O_2_ during the first ten million years of its existence. This is frontally opposed to the idea that Proxima Centauri b could harbor a vast ocean. The addition of frequent solar storms and intense magnetic activity also places it far from being the ideal place to shelter any kind of life (Anglada-Escude et al., 2016).

Some recent researches have found other exoplanets, such as LHS 1140b, transiting a small cool star (LHS 1140), within the liquid water habitable zone (Dittmann et al., 2017). Moreover, recent observations revealed that seven planets similar to Earth rotate around their host star named TRAPPIST-1 (Gillon et al., 2017). Their surface temperatures are low enough to enable the existence of liquid water (Stevenson, 1999; Kopparapu et al., 2013; Leconte et al., 2013). Upcoming observations with large ground and space-based telescopes may help to illuminate the intriguing environment of our nearest exoplanetary neighbor.

## Environmental Life-Limiting Characteristics of Terrestrial Glaciers and ice Sheets

Several reviews have been published with the aim of evaluating the key sites that are contributing most to our understanding of potential extraterrestrial habitable environments in the solar system (Preston and Dartnell, 2014). Studies aimed at detecting life beyond Earth have inevitably been centered on Earth life because this is the only example of life that we know. Therefore, it is necessary to study environments on Earth that are analog to those in other planets and satellites. It is also important to consider that many terrestrial environments in which life exists today may not be adequate for the life that originated 1000s of years ago.

Several common habitability conditions have been defined (Priscu and Hand, 2012), but the environmental conditions of the extraterrestrial systems we know are very different from each other and very different from those on Earth. A potentially habitable location needs to meet a number of requirements. The abovementioned requirements -solvent, energy, essential elements and physicochemical conditions- must be present in limited quantities. However, which are those limits that determine life? To know the limits of life on Earth, we have to consider the extremophile organisms. These can be defined as organisms living in physical or geochemical conditions that are incompatible with the life of most organisms. Thus, they live to the limit. These organisms (usually microorganisms, as they are simpler) collectively define the physiochemical limits to life. Extremophiles are able to live in harsh environments such as volcanic hot springs, glaciers, extremely salty environments, in waters having a pH as low as 0 or as high as 12, or in the deep sea under extreme pressure (Pikuta et al., 2007). Interestingly, these prokaryotes do not only tolerate their particular environmental extreme conditions, but they require it in order to grow (Madigan et al., 2012). **Table 2** summarizes some examples among extremophiles, the especial conditions these microorganisms support, the type of habitats in which they reside, and the extraterrestrial environments to which they are analog.

**Table 2:** Extremophiles on Earth with similar environments to the icy worlds of the solar system.

Extremophile	Conditions	Earth habitat	Analog environments	Examples of microorganisms
Psychrophiles	Low temperature	Glaciers, sea ice	Ice shells of Europa and Enceladus; poles of Mars	*Psychrobacter*^b^
Acidophiles	Low pH	Mines, volcanoes	Surface of Mars	*Acetobacter*^b^
Alkaliphiles	High pH	Soda lakes	Ocean of Enceladus	*Bacillus firmus*^b^
Halophiles	High salinity	Salterns, sea ice inclusions	Subsurface oceans of Europa, Titan and Enceladus	*Halobacterium*^a^
Barophile or Piezophiles	High pressure	Deep ocean	Ocean floors of Europa	*Moritella*^b^
Xerophiles	Low water activity	Deserts, rock surfaces	Surface of Mars	*Bacillus megaterium*^b^
Radiotolerant	High radiation	Nuclear reactor water	Surface of Europa	*Deinococcus*^b^


*^a^Archaea. ^b^Bacteria*.

On Earth, three environments maintain very low temperatures throughout the year: poles, oceans and glaciers. Considering that a great diversity of microorganisms belonging to the three main domains (Bacteria, Eucarya, and Archaea) has been discovered inhabiting glaciers (Garcia-Lopez and Cid, 2017), they have been considered biomes that should be recognized as such in their own right (Hodson et al., 2008; Anesio and Laybourn-Parry, 2012).

The inhabitants of the glaciers have to be polyextremophiles, due to the diverse extreme conditions in which they live (**Table 2**). Most of the microorganisms isolated from cold environments are psychotolerant (also called psychrotrophs) and psychrophiles. Psychotolerant organisms can grow at temperatures close to 0°C but have their optimum growth temperature at about 20°C. However, psychrophiles have their optimal growth temperature at 15°C or less (Cavicchioli et al., 2002). In addition to the low temperatures, microorganisms from glaciers generally tolerate high solar radiation -radiophiles- (Martin-Cerezo et al., 2015), scarce availability of water -xerophiles- (Imshenetsky et al., 1973), and sometimes they can also bear extremely acidic media -acidophiles- (Cid et al., 2010).

## Microbial Communities Living in Glaciers and ice Sheets

All the organisms are composed of nearly the same macromolecules, but the environments and physical–chemical conditions in which they live are very different. Some organisms can cope with very extreme conditions, and the knowledge of this versatility of life on Earth can help the understanding of a hypothetical life in other worlds (Des Marais et al., 2008).

**FIGURE 2:**
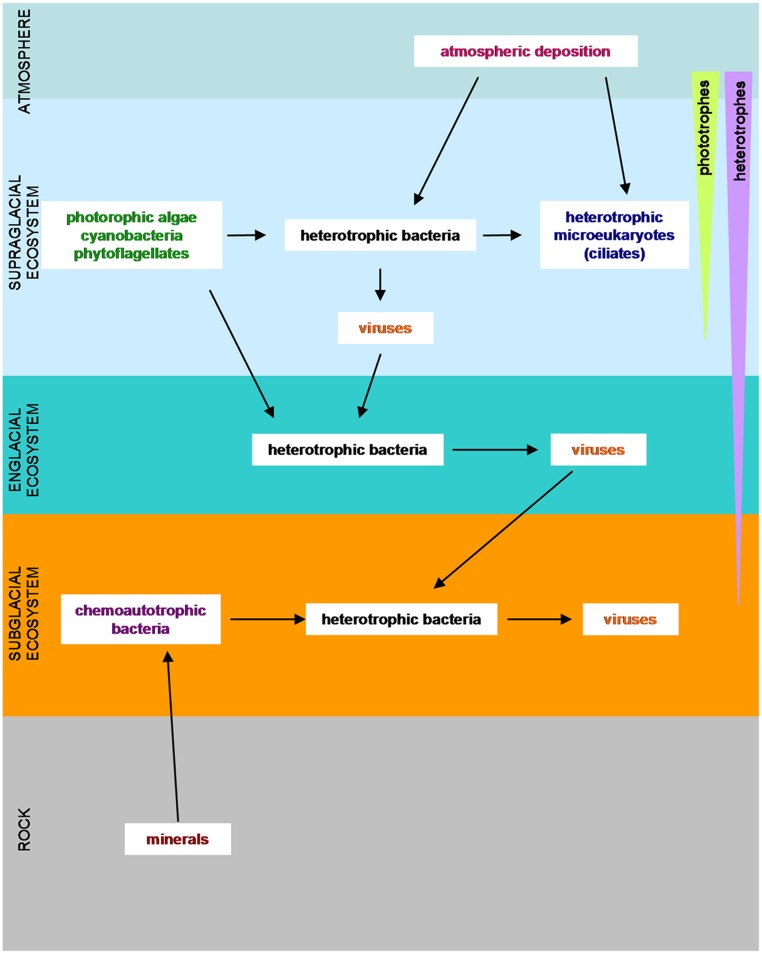
Overview of the microorganisms in glaciers and ice sheets with their food requirements. (Adapted from Hodson et al., 2008; Anesio and Laybourn-Parry, 2012; Garcia-Lopez and Cid, 2017).

The discovery of cold-tolerant microorganisms in glaciated and permanently frozen environments has broadened the known range of environmental conditions that support microbial life. In glaciers and ice sheets, three different ecosystems have been considered: the supraglacial ecosystem, the subglacial system and the englacial ecosystem (Hodson et al., 2008; Boetius et al., 2015) (**Figure 2**). These three ecosystems differ in terms of their solar radiation, water content, nutrient abundance and redox potential (Hodson et al., 2008).

The supraglacial ecosystem is characterized by the absorption of solar radiation, which causes ice melting yielding liquid water. Meltwater dissolves nutrients from adjacent rocks, and even directly from the atmosphere (Tranter et al., 1993). This facilitates the growth of microbial colonies (Maccario et al., 2015) of autotrophic microorganisms such as microalgae and diatoms. The supraglacial surface is also populated by chemolitotrophic bacteria, which feed on inorganic sand particles, by heterotrophic bacteria and by microeukaryotes (Mieczen et al., 2013). Cryoconite holes, cylindrical melt holes in a glacier surface (Fountain et al., 2008), have recently gained attention because they may be a refuge similar to the one that could be inhabited by the microorganisms in the frozen worlds (Vincent and Howard-Williams, 2000; Nisbet and Sleep, 2001) or on planet Earth during the glaciations (Hoffman et al., 1998; Priscu and Christner, 2004).

The englacial ecosystem presents a minor impact upon nutrient dynamics (Hodson et al., 2008), but it is the most analogous to the environment that could be found in icy worlds. Biomass is low in the englacial ecosystem, and microorganisms live in places that protect them against radiation and dehydration (Aerts et al., 2014). Inside glacier ice, bacteria live at clay particles or at grain boundaries, and other interstices like triple point junctions, brine channels and gas bubbles. Mineral substrates provide nutrients and a supply of water for microorganisms (Price, 2007). Englacial ecosystems are mainly inhabited by chemoautotrophs; but they can also be heterotrophic bacteria that feed on solubilized organic products. At great depth, anaerobic respiration and methanogens can take place (Tung et al., 2005; Price, 2007; Hodson et al., 2008).

The subglacial ecosystem is composed of sediments and bedrock. These environments are isolated by the upper deposits of glacial ice, which decreases temperature fluctuations and make them suitable for microbial life (Sharp et al., 1999). For example, beneath a High Arctic Glacier, aerobic chemoheterotrophs and anaerobic nitrate reducers, sulfate reducers, and methanogens have been identified (Skidmore et al., 2000; Phillips and Parnell, 2006; Harrold et al., 2016). Underneath the glaciers, microorganisms can persist in a quiescent way for years at subfreezing temperatures (Sharp et al., 1999; Skidmore et al., 2000). When conditions are favorable, and partial melting of the ice occurs by a temperature rise, microorganisms may be reactivated. The presence of liquid water, favored by the high pressure exerted by overlying layers of ice is a good solvent for the organic and inorganic nutrients from dissolved particulate material and gasses. Some ingredients necessary for life such as the organic carbon, derive from soil and decaying plants overridden by glacial advance. There is evidence that some redox reactions like sulfide oxidation (Raiswell, 1984), denitrification (Tranter et al., 1994), iron reduction (Nixon et al., 2017) and oxidation of organic carbon (Fairchild et al., 1993) can occur at subglacial sediments. Furthermore, some microbial communities are able to live nearly isolated from the atmosphere in subglacial environments, thanks to their chemolithotrophic metabolism (Boyd et al., 2014).

**FIGURE 3:**
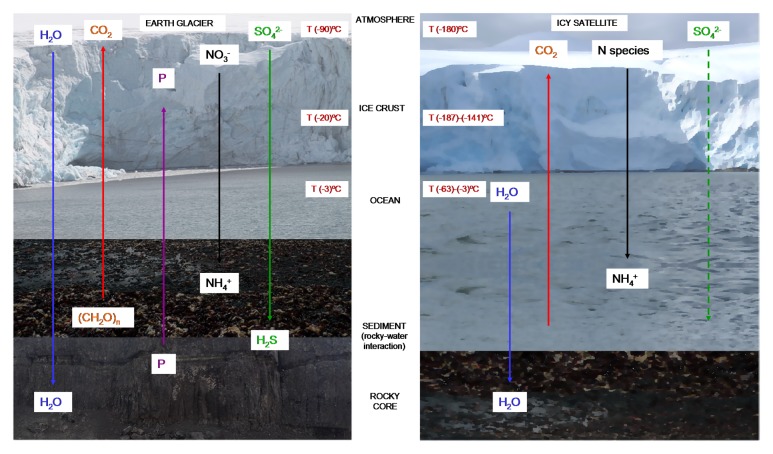
Stratification of nutrients and temperatures in cold habitats. Microbial metabolism in terrestrial glaciers (left) is driven by coupling surface oxidants with reductants associated with the subglacial rocks (Rankin et al., 1999; Hodson et al., 2008; Murray et al., 2012). The same mechanism could be possible in icy worlds (right), in which come of this nutrients have been detected (Cockell et al., 2016). Temperature on the surface of Europa is around -180°C, but the interior ocean can have a much warmer temperature that reaches -3°C (Spencer et al., 1999). At the coldest point of the Earth’s surface (Base Vostok, Antartica) temperatures can reach -90°C, but inside the Antarctic Lake Vostok the temperature is also around -3°C (Priscu and Hand, 2012).

Subglacial water can be very abundant in subglacial rivers and lakes such as the Blood Falls (Mikucki et al., 2009) or Lake Vostok (Christner et al., 2006; Rogers et al., 2013) in Antarctica. These environments have been studied with great interest because they can be considered analogs of the underground oceans of some icy satellites (**Figure 3**). High pressures and high salinity of water running through subsurface or ice depositions lower the freezing point of water and thus provide liquid water available for biochemical processes (Aerts et al., 2014). These subglacial ecosystems harbor diverse communities of heterotrophic and lithotrophic microorganisms over extended periods of time. They are dominated by bacteria and viruses in basal bedrock and subglacial lakes, and they also contain, metabolically active archaeal, bacterial and fungal species (Butinar et al., 2007). At this depth, a diversity of metabolic mechanisms may be expected, but processes such as serpentinization (Michalski et al., 2013) could possibly provide energy for microorganisms in subglacial ecosystems. For instance, the permanent ice covered Lake Vida (Antarctica) contains a cryogenic, aphotic and anoxic ecosystem in which active bacteria live under very high levels of reduced metals, ammonia, dissolved organic carbon; as well as high concentrations of oxidized species of nitrogen and sulfur (Rankin et al., 1999; Murray et al., 2012).

In icy satellites such as Europa, a similar environment could exist. Liquid water could be present in the subsurface ocean, containing materials derived from the original formation of the satellite (Dalton et al., 2010). In addition, new compounds may have been produced by chemical reactions in the interior, reaching the surface by endogenic processes such as cryovolcanism or extensional tectonics. Additional material may be brought by comets or meteorites (Dalton et al., 2010). N species could come from the surface crust; and CO_2_, SO_4_^2-^ and organic compounds could be expected due to meteoritic delivery (Cockell et al., 2016). Other compounds such as HCN have been detected in other icy satellites such as Enceladus, Triton and Titan (Dalton et al., 2010; Cockell et al., 2016; Marin-Yaseli et al., 2017) (**Figure 3**).

### Molecular Mechanisms of Adaptation to Cold Environments

Inside glaciers and ice sheets, ice veins provide a liquid microenvironment, which may serve as a habitat for microbial life (Thomas and Dieckmann, 2002). However, microorganisms in the ice veins support a variety of physiochemical stresses including low water activity, low pH, reduced solute diffusion rates and membrane damaging due to ice crystal formation. To grow efficiently at low temperatures, microorganisms have developed complex structural and functional strategies for their adaptation (D’Amico et al., 2006). The study of these adaptation strategies aims to identify the limits of life at these temperatures (**Table 3**). Adaptations include: (i) the synthesis of catalytically efficient enzymes that are functional at low temperatures with a high efficiency (Feller and Gerday, 2003); (ii) the synthesis of specialized unsaturated fatty acids in the cell membrane to increase its fluidity (Los and Murata, 2004; Nichols et al., 2004); (iii) the production of extracellular polymeric substances (Krembs et al., 2011) which affect ice crystal structure and allow the cell to protect itself from frostbite (e.g., sugars, polysaccharides, antifreeze proteins) (Alcazar et al., 2010; Garcia-Descalzo et al., 2012a); (iv) the synthesis of certain proteins that allow synthesizing others at low temperatures (Garcia-Descalzo et al., 2011); (v) the reorganization of protein networks (Garcia-Descalzo et al., 2014); (vi) the use pigments to obtain energy, stress resistance and for ultraviolet light protection (Garcia-Lopez and Cid, 2016). For instance, colored glacier surfaces are caused by snow algae or melanized fungi. Additionally, some cold-adapted bacteria produce pigments such as xanthins, carotenes and cytochromes (Garcia-Descalzo et al., 2014).

**Table 3:** Mechanisms of adaptation to extreme environments.

Extremophile	Challenges	Mechanisms of adaptation
Psychrophiles	Reduced enzyme activity Decreased membrane fluidity and altered transport Decreased rates of transcription, translation and cell division Protein cold denaturation, inappropriate protein folding	Active enzymes at low temperature Cold shock proteins Fats that allow membrane fluidity Antifreeze compounds
Acidophiles	Alteration in the cellular membrane and transport	H^+^ transport pumps Active enzymes at low pH
Alkaliphiles	Alteration in the cellular membrane and transport	Active enzymes at high pH
Halophiles	Water loss and desiccation	Synthesis of compounds such as betaine, glycerol, etc. Active enzymes at high salinity
Barophile or Piezophiles	Decreases the ability of the subunits of proteins to interact Protein synthesis, DNA synthesis, and nutrient transport are sensitive to high pressure	Higher proportion of unsaturated fatty acids in cytoplasmic membranes Special membrane proteins
Xerophiles	Water loss and desiccation	They live inside rocks to use water condensation
Radiotolerant	DNA mutations	Multiple copies of DNA Various mechanisms of DNA repair


In recent years, a few studies have been published on the recovery of live microorganisms from ancient ice, where they have survived for 100s of millions of years (Bidle et al., 2007). The endurance of these microorganisms depends on their ability to persist in a dormant, metabolically inert state. This means that glaciers and ice sheets are inhabited by microorganisms, which maintain active biochemical processes to preserve their cellular integrity and a minimal metabolism (Dieser et al., 2013). The long-term survival does not imply a very active functional metabolism, but it is at least necessary that the microorganisms can recover the damaged molecules, such as the DNA that breaks because of background radiation in the permafrost (Johnson et al., 2007).

### Detection of Microbial Activity at Subzero Temperature

Due to techniques such microscopy, and more recently DNA sequencing methods, a great biodiversity of prokaryote and eukaryote microorganisms have been discovered inhabiting glaciers and ice sheets (**Table 4**).

**Table 4:** Evidence for activity in microorganisms at subzero temperatures.

Sample	Temperature (°C)	Activity	Technique	Reference
Lake microbial community	0	Carbon fixation Fermentation Methanogenesis Methane oxidation CO oxidation Nitrogen assimilation Denitrification Nitrogen fixation Sulfate reduction Sulfide oxidation	Proteomics	Lauro et al., 2011
Antarctic sea bacterioplankton	-0.92	Ammonia oxidation Reverse tricarboxylic acid cycle	Proteomics	Williams et al., 2012
*Methanococcoides burtonii*	-2	EPS production Oxidative stress Quality control of protein folding	Proteomics	Williams et al., 2011
Greenland sea ice	-4	Morphology and motility	Microscopy	Lindensmith et al., 2016
Ice-sealed Antarctic lake	-13	Macromolecular Synthesis	^3^H-leucine incorporation	Murray et al., 2012
Basal glacier ice	-15	Macromolecular Synthesis	^3^H-thymidine and ^3^H-leucine incorporation	Doyle et al., 2013
Snow	-17	Macromolecular Synthesis	^3^H-thymidine and ^3^H-leucine incorporation	Carpenter et al., 2000
Sea ice	-20	Respiration	5-cyano-2,3-ditolyl tetrazolium chloride reduction	Junge et al., 2004
Permafrost	-39	Respiration	[^14^C]Glucose uptake	Panikov et al., 2006
*Psychrobacter cryohalolentis* K5	-80	Metabolism	ATP and ADP levels	Amato and Christner, 2009
Environmental isolates from sea ice	-196	Protein synthesis	^3^H-leucine incorporation	Junge et al., 2006


Morphology and motility of microbial cells observed by *in situ* microscopy have been used as a technique for the detection of biosignatures in the liquid brines that persist in ice (Lindensmith et al., 2016; Nadeau et al., 2016). Microbial metabolism in glaciers and ice sheets is driven by coupling surface oxidants with reductants associated with the subglacial rocks (**Figure 3**). The incorporation of radio labeled compounds has been used to detect cellular respiration (Panikov et al., 2006), to investigate DNA, lipid or protein synthesis and to reveal CH_4_ production (Doyle et al., 2012, 2013; Murray et al., 2012). Using these methods, it has been shown that snow and firn contain bacteria able to maintain low rates of DNA and protein synthesis, and different microorganisms able to metabolize at subzero temperatures (-12°C to -17°C) (Carpenter et al., 2000; Siddiqui and Cavicchioli, 2006). Other analytical techniques such as the measurement of ATP and ADP concentrations demonstrated the existence of metabolism in the psychrophilic bacteria *Psychrobacter cryohalolentis* K5 cultured at -80°C (Amato and Christner, 2009); although, the lower limit of temperature at which microbial activity has been detected in laboratory culture seems to be -196°C (Junge et al., 2006). Additionally, measurement of nitrate and ammonium has been performed to detect N mineralization and nitrification. The reduction of some chemical compounds such as 5-cyano-2, 3-ditolyl tetrazolium chloride (CTC) has been used to detect cellular respiration (Junge et al., 2004).

Recently, the diversity of life forms and mechanisms of adaptation to extreme conditions, and the characterization of the specific signs of their biological activity on glaciers have been investigated with other approaches such as Fourier Transform Infrared Spectroscopy (FTIR), Raman spectroscopy, and “omics” technologies (genomics, transcriptomics, proteomics or metabolomics).

Specifically, proteomic techniques have identified proteins that are active at cold temperatures, showing that microorganisms are alive and metabolically active inside glaciers (**Table 4**). For example, in the cold-adapted microorganism *Methanococcoides burtonii* transcription and translational mechanisms are compromised at -2°C, which enables very low growth rates (Williams et al., 2011). The advantage of using proteomic techniques over other analytical techniques, is that proteomics can simultaneously detect various activities of microorganisms by the identification of specific proteins involved in each cellular process. When combined with genomic techniques, proteomics can identify the activity of complete microbial communities. Another advantage of studying proteins is that they are molecules that actually work in the cell. By determining which proteins have been synthesized by microorganisms, metaproteomics enables the reconstruction of microbial processes and metabolic pathways that are central to the functioning of the ecosystem (Williams et al., 2012). This is especially interesting in the study of microbial communities from glaciers and ice sheets, where cell motility is lower than it is in aqueous media such as lakes and oceans (Lauro et al., 2011). Therefore, proteomics presents an attractive alternative for the direct identification of biosignatures in analog environments of icy worlds (Garcia-Descalzo et al., 2012b; Martin-Cerezo et al., 2015).

It has also been shown that microbial proteins take part in complexes, which are modulated by the environment (Garcia-Descalzo et al., 2014). Therefore, the molecular machinery in cold-adapted microorganisms is very adaptable through the interaction network they establish. Proteins that take part in networks are dependent on environmental conditions. For instance, it has been demonstrated that the protein HSP90 and HSP90-associated proteins from microorganisms inhabiting analogous environments conserve similar HSP90 interactors in opposition to phylogenetically closely related microorganisms living in different environments (Garcia-Descalzo et al., 2011).

## Concluding Remarks

Icy worlds have attracted a remarkable attention in the search for habitability beyond Earth.

Here we have compared the environmental characteristics of icy worlds and the environmental characteristics of terrestrial glaciers and ice sheets in order to address some interesting questions. Firstly, which are the characteristics of habitability known for the frozen worlds, and which could be compatible with life? Secondly, what are the environmental characteristics of terrestrial glaciers that can be life-limiting, and which are the microbial communities that can live in them? And finally, taking into account all of these observations, could any of these planets or satellites meet the conditions of habitability? After comparing the characteristics of habitability of icy worlds and Earth glaciers, it can be concluded that the icy worlds of the solar system most likely to harbor life are Europa and Enceladus (**Table 1**). Other icy worlds could also contain a water ocean below the surface, but it would be included between layers of ice (Dalton et al., 2010). Nevertheless, Europa and Enceladus are the only two recognized moons where liquid water could be in contact with rocks. This conclusion is supported by the recent observations made by the Cassini spacecraft, which found molecular hydrogen in the Enceladus’ plume, suggesting the possible existence of hydrothermal processes (Waite et al., 2017).

In the pursuit of life in other planets, it is widely recognized that the presence of liquid water is requirement for habitability. And it has been demonstrated not only that life is possible inside glaciers and ice sheets, but also that ice could constitute a shelter to protect microorganisms from solar radiation. The existence in Earth glaciers of microbial communities that maintain active biochemical routes in englacial and subglacial ecosystems can broaden the scenarios in which life might be possible.

These findings open new questions to research: (i) as the studies of enzymatic activity at very low temperatures (up to -196°C) have mostly been carried out in the laboratory, could these metabolic activities be detected *in situ* in the coldest regions of Earth?, (ii) are we using the best techniques to detect biosignatures on icy worlds?, (iii) what will we find in upcoming missions to Europa and Enceladus?, (iv) are there other new potential candidate worlds to be habitable?, (iv) what characteristics of habitability can be found in exoplanets? The future space exploration of these icy worlds will be challenging.

## Author Contributions

CC: wrote the manuscript, reviewed the literature on microbiology of glaciers, and edited figures. EG-L: reviewed the literature on astrobiology and habitability and wrote the first part of the manuscript.

## Conflict of Interest Statement

The authors declare that the research was conducted in the absence of any commercial or financial relationships that could be construed as a potential conflict of interest.
